# AAV-8 and AAV-9 Vectors Cooperate with Serum Proteins Differently Than AAV-1 and AAV-6

**DOI:** 10.1016/j.omtm.2018.08.001

**Published:** 2018-08-08

**Authors:** Jérôme Denard, Jérémy Rouillon, Thibaut Leger, Camille Garcia, Michele P. Lambert, Graziella Griffith, Christine Jenny, Jean-Michel Camadro, Luis Garcia, Fedor Svinartchouk

**Affiliations:** 1 Généthon, Evry, France; 2 Institute Jacques-Monod, Paris, France; 3 Department of Pediatrics, Division of Hematology, Children’s Hospital of Philadelphia, Philadelphia, PA, USA; 4 Université de Versailles St-Quentin, INSERM U1179, Montigny-le-Bretonneux, France; 5 SQY Thérapeutics, UVSQ, UFR des Sciences de la Santé, 2 Avenue de la Source de la Bièvre, 78180 Montigny-le-Bretonneux, France

**Keywords:** adeno-associated vectors, blood proteins, platelet factor 4, virus-host interactions

## Abstract

Under intravenous delivery, recombinant adeno-associated vectors (rAAVs) interact with blood-borne components in ways that can critically alter their therapeutic efficiencies. We have previously shown that interaction with human galectin 3 binding protein dramatically reduces rAAV-6 efficacy, whereas binding of mouse C-reactive protein improves rAAV-1 and rAAV-6 transduction effectiveness. Herein we have assessed, through qualitative and quantitative studies, the proteins from mouse and human sera that bind with rAAV-8 and rAAV-9, two vectors that are being considered for clinical trials for patients with neuromuscular disorders. We show that, in contrast to rAAV-1 and rAAV-6, there was a substantial similarity in protein binding patterns between mouse and human sera for these vector serotypes. To establish an *in vivo* role for the vector binding of these sera proteins, we chose to study platelet factor 4 (PF4), which interacts with both vectors in both mouse and human sera. Experiments using PF4-knockout mice showed that a complete lack of PF4 did not alter skeletal muscle transduction for these vectors, whereas heart transduction was moderately improved. Our results strongly support our position that the impact of serum proteins on the transduction properties of rAAV-8 and rAAV-9, already observed in mouse models, should be similar in human preclinical trials.

## Introduction

Adeno-associated virus (AAV)-derived recombinant vectors (rAAV) are attracting significant attention as promising tools for a wide range of applications in the field of gene therapy. Cell transduction mechanisms with rAAV have been studied in detail. Those studies have identified a number of cellular receptors for virus entry, as well as many aspects of the intracellular trafficking of their payloads to the nucleus. Protein classes having specific post-translational modifications, such as alpha-2,3 and alpha-2,6 sialic acids, N-linked glycoproteins, or heparin sulfate proteoglycans, are the primary cell receptors for rAAV uptake.^1^, ^2^, ^3^ These post-translational modifications are so common among mammals that researchers initially assumed that rAAV efficiency would be similar across species lines, such that data obtained from animal models would be predictive of the human situation. This optimism, however, was tempered by subsequent studies showing that rAAV-3 could efficiently transduce human hepatocytes through the human hepatocyte growth factor receptor (HGFR), but had no such uptake mechanism in murine hepatocytes.^4^, ^5^ Moreover, many studies have been completed using cells grown in culture,^6^, ^7^, ^8^ without taking into account the likely disruptive interactions of rAAV with actual components of more complex human body fluids.

This consideration is a crucial issue in the case of systemic delivery of vectors in humans via intravenous transfusions. Indeed, recent studies have shown that rAAV interactions with blood proteins are significantly vector-serotype and species-sera specific. As an example, human and dog galectin 3 binding protein (G3BP) interacts with serotype rAAV-6 and decreases its transduction efficiency, but mouse and monkey G3BP do not.^9^ The same applies to mouse C-reactive protein (CRP); it binds with rAAV-1 and rAAV-6, improving skeletal muscle transductions by more than 10-fold in mice, but human CRP does not react with these two serotypes.^10^

Given that knowing about and taking into consideration these critical species-specific concerns is essential for further improvements in rAAV-driven therapeutics, we have undertaken studies to precisely identify the patterns of serum proteins reacting with rAAV-8 and rAAV-9, two vector serotypes that are currently under widespread clinical development. By using an assay that is comprised of direct trypsin digestion of serum proteins co-precipitated with immobilized vectors and Orbitrap mass spectrometry peptide analysis, we were able to exhaustively identify and quantify the serum proteins interacting with these important vectors. We show that, in contrast to rAAV-1 and -6, which preferentially interact with one major serum protein (CRP in murine sera and G3BP in human sera), rAAV-8 and -9 interacted with a larger and more diverse spectrum of proteins in mouse and human sera.

Importantly, we observed a high similarity in the patterns of bound proteins between mouse and human. As stated, rAAV-8 and -9 do not bind one predominant protein, instead they bind to up to 30 different proteins, at rates ranging from 0.1% to 25% of the total amounts of bound proteins. Second, there were nine proteins bound to rAAV-8 in common between mouse and human sera. Quantitative estimation of proteins bound to rAAV-8 demonstrated that these nine proteins comprised 50% of the bound protein in mouse sera and 40% in human. Similarly, there were six proteins in common between mouse and human sera that bound to rAAV-9; they comprised 86% and 51% of the bound proteins, respectively.

Next, we assessed whether these proteins might have a functional impact on vector transduction comparable with that of murine CRP or human G3BP on the efficacy of rAAV-1 and rAAV-6, by evaluating the functional role of platelet factor 4 (PF4). This protein was found to have the highest level of vector binding in human and mouse sera for both serotypes (AAV-8 and -9), although comprising only roughly 15% of the total bound proteins. Using PF4-knockout (KO) mice and PF4-KO mice expressing human PF4 (huPF4), we showed *in vivo* that serum lacking mPF4, or huPF4, did not alter skeletal muscle transduction, even though the efficacy of the level of heart transduction was improved by 2- to 3-fold for both vectors. Our results strongly support our position that the impact of serum proteins on the transduction properties of rAAV-8 and rAAV-9, already observed in mouse models, should be similar in human preclinical trials.

## Results

### Identification of Serum Proteins Interacting with rAAV

To accurately identify the proteins interacting with rAAV-8 and -9, we adapted the technology of a vector-protein binding assay, in which serum proteins bound to immobilized rAAV particles are digested with trypsin and the resulting peptides are identified on an Orbitrap mass spectrometry (MS) instrument. This was followed by estimation of the relative abundance of each protein by a label-free quantification approach.^11^ Only proteins identified as present by three or more peptides, and a Mascot score exceeding 70, were given further analysis. We used defined criteria to discriminate proteins specifically bound to the rAAV particles from any non-specifically bound proteins, i.e., those proteins bound adventitiously to the bead support used for rAAV immobilization. These criteria were: (1) specificity (the quantity ratio of a protein bound to bead-immobilized rAAV that bound to empty beads), which had to be higher than 2; and (2) reproducibility, only proteins detected in at least 50% of the experiments were given further analysis (five to eight independent experiments were performed for each serotype and serum).

We validated our assay by reanalyzing proteins captured by the well-studied rAAV-6 in the presence of human or mouse sera (Table 1). In agreement with previous studies, human G3BP matched both of our criteria, with a >100 “specificity” in six of six experiments. The same was true of murine CRP, which displayed a >100 “specificity” in all eight experiments. Remarkably, although human G3BP and murine CRP were the major proteins bound to rAAV-6, representing more than 90% of the quantity of bound proteins, our assay made it possible to now identify additional proteins not detected using previous assays.^9^, ^10^ Minor amounts of vitronectin, prothrombin, fibronectin, and lipopolysaccharide binding protein were captured from human serum by rAAV-6 (Table 1). Likewise, in more than 50% of the experiments using mouse serum, rAAV-6 could also bind small amounts of vitronectin, prothrombin, and complement C1q subcomponent subunits A and B (Table 1).

**Table 1:** Proteins in Human and Mouse Serum Interacting with rAAV-6

Description	% of Bound Proteins^a^	n^b^
Human Serum		

Galectin-3-binding protein	96.08	6
Vitronectin	2.33	4
Prothrombin	0.86	3
Fibronectin	0.37	3
Lipopolysaccharide-binding protein	0.13	3

Mouse Serum		

C-reactive protein	90.68	8
Vitronectin	4.68	4
Prothrombin	2.80	4
Complement C1q subcomponent subunit B	1.50	6
Complement C1q subcomponent subunit A	0.47	5

a The sum of protein quantities meeting the selection criteria for rAAV-6 was considered to be 100%; the relative quantity of each protein is expressed as a % of the sum.

b n is the number of experiments where the protein was detected. Six independent experiments were performed with human serum and eight with mouse serum.

**Figure 1:**
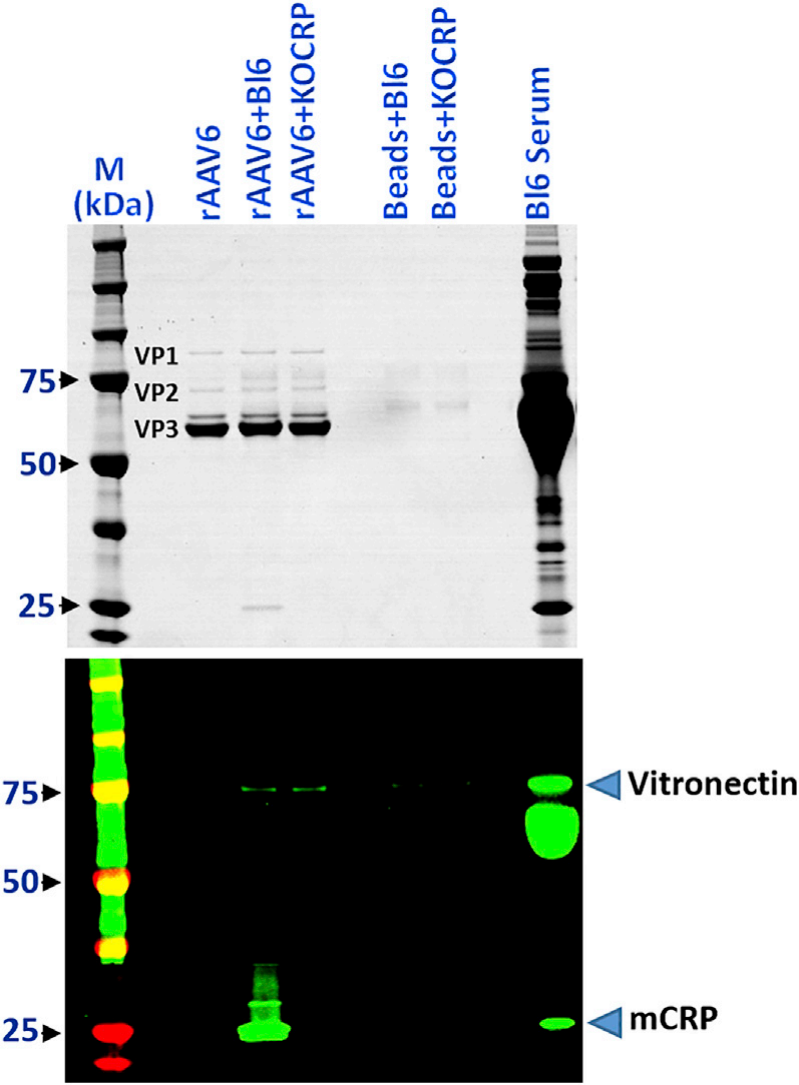
CRP and Vitronectin Possess Independent Binding Sites on rAAV-6 Serum proteins bound to rAAV-6 were co-precipitated from C57BL/6 and C57BL/6 KO-CRP mouse sera and separated on gels, as described in the Materials and Methods. Upper panel: Coomassie staining (VP1, VP2, VP3 indicate the position of the respective vector proteins); lower panel: western blot analysis of CRP and vitronectin binding to rAAV-6. From left to right: M, molecular weight (MW) markers; rAAV6, immobilized vector without incubation with serum; rAAV6 + Bl6 and rAAV6 + Bl6 KO-CRP, deposition of proteins onto immobilized vectors after incubation in serum from C57BL/6 and from C57BL/6 KO-CRP mice, respectively; Beads + Bl6 and Beads + Bl6 KOCRP, empty beads incubated with serum from C57BL/6 and C57BL/6 KO-CRP mice, respectively; Bl6 serum: 0.5 μL of C57BL/6 serum.

To determine whether minor and major proteins might share the same binding sites on rAAV-6, we compared the respective binding levels of vitronectin in sera from either wild-type C57BL/6 mice or C57BL/6 CRP-KO mice.^10^ Figure 1 clearly shows that the absence of CRP did not increase vitronectin capture, indicating that there was neither competition nor co-operation between the two ligands, CRP and vitronectin.

### Serum Proteins Interacting with rAAV-8 and rAAV-9 in Mouse Serum

The list of proteins interacting with rAAV-8 and rAAV-9 in mouse serum is presented in Table 2. Because we did not observe any differences in patterns of identified proteins for vectors prepared either by HEK293 transfection or by Sf9-Baculovirus, the origin of vectors is not indicated in Table 2. Unlike rAAV-6, where CRP accounted for more than 90% of all bound protein, no majority protein (greater than 50%) was identified for rAAV-8 and rAAV-9. Overall, 15 proteins met the selection criteria for rAAV-8, and 12 proteins for rAAV-9, with 7 proteins being in common to both serotypes. These seven in-common proteins accounted for roughly 50% of the bound proteins in the case of rAAV-8 and more than 60% for rAAV-9. Interestingly, the top four proteins (PF4, complement C3, glycosylation-dependent cell adhesion molecule 1 [GLCM1], and vitronectin) accounted for the 50% of the bound protein in the case of rAAV-8, and the top four proteins for rAAV-9 represented 70% of the bound protein (Table 2).

**Table 2:** Proteins in Mouse Serum Interacting with rAAV-8 and rAAV-9

rAAV-8			rAAV-9		
Description	% of Bound Proteins^a^	n^b^	Description	% of Bound Proteins^a^	n^b^
Platelet factor 4^c^	15.7	4	Histidine-rich glycoprotein	25.2	7
Complement C3	14.2	4	Platelet factor 4^c^	19.2	4
Glycosylation-dependent cell adhesion molecule 1	12.0	7	Vitronectin^c^	18.5	7
Vitronectin^c^	10.3	8	Prothrombin^c^	6.9	7
Complement C1q subcomponent subunit A^c^	5.9	7	Inter-alpha-trypsin inhibitor, heavy chain 4^c^	6.5	7
Complement C1q subcomponent subunit B	5.5	7	Thrombospondin-1^c^	6.5	5
Thrombospondin-1^c^	5.3	6	Clusterin	5.0	7
Complement C1q subcomponent subunit C	5.2	5	Murinoglobulin-1	4.9	5
Fibronectin	4.9	6	Glutathione peroxidase 3	4.7	7
Clusterin^c^	4.6	7	Glia-derived nexin	1.2	7
Prothrombin^c^	4.5	7	Coagulation factor V	0.8	6
Complement C4-B	4.0	4	Complement C1q subcomponent subunit A^c^	0.6	4
Metalloproteinase inhibitor 3	3.9	6	–	–	–
Inter-alpha-trypsin inhibitor, heavy chain 4^c^	3.6	7	–	–	–
Glia-derived nexin	0.7	6	–	–	–
Common with AAV9	49.8	–	Common with AAV8	63.2	–

a The sum of protein quantities meeting the selection criteria for the vectors was considered to be 100%, and the relative quantity of each protein is expressed as a % of the sum. Proteins are arranged in the order of decreasing quantities.

b n is the number of experiments where a protein was detected. Eight independent experiments were performed with rAAV-8 and seven with rAAV-9.

c Proteins are common between rAAV-8 and rAAV-9.

**Figure 2:**
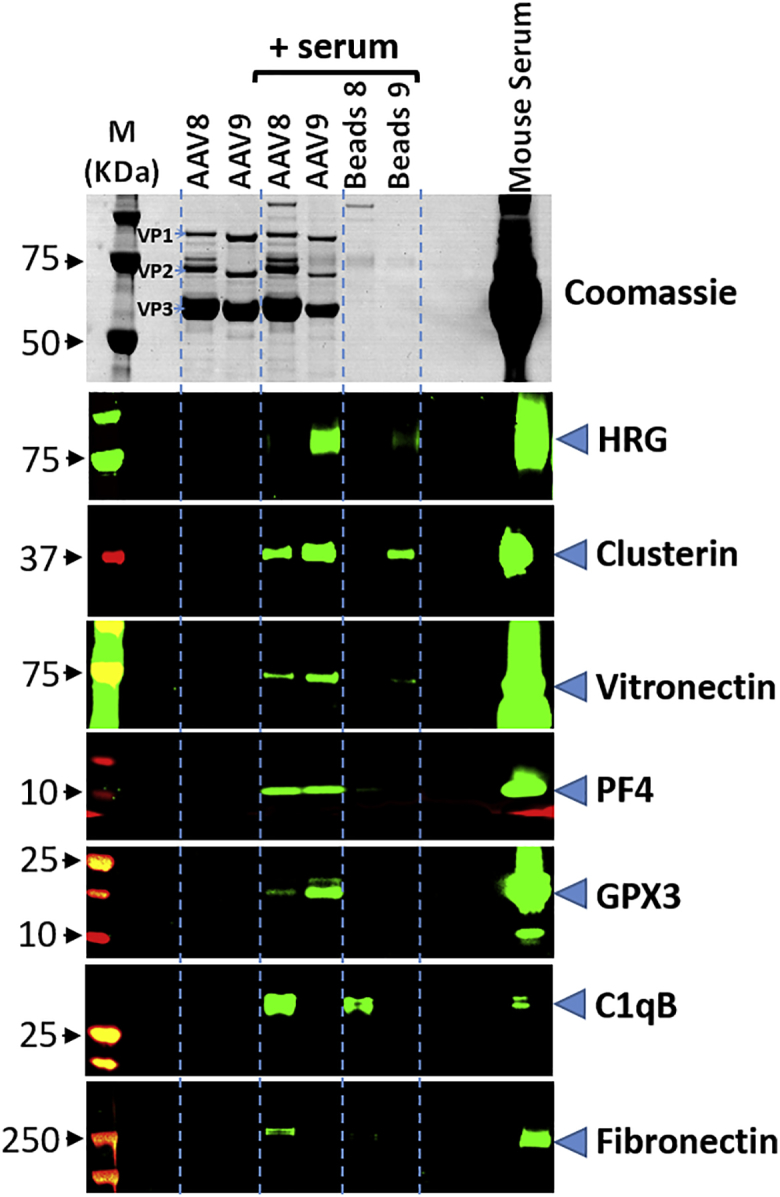
Western Blot Analysis of Proteins Binding to rAAV-8 and rAAV-9 in Mouse Serum Upper panel: Coomassie staining (VP1, VP2, and VP3 indicate position of the respective vector proteins); lower panel: western blot analysis of selected proteins binding to rAAV-8 and rAAV-9. From left to right: M, molecular weight (MW) markers; AAV8, AAV9, respective immobilized vectors without incubation with serum; AAV8, AAV9 (serum), respective immobilized vectors after incubation with serum; beads 8, beads 9 (serum), empty beads used for the respective serotype immobilization after incubation with serum; mouse serum, deposition of 0.8 μL of serum.

Where antibodies were commercially available, we verified protein binding to the vector by western blot analysis (Figure 2). Importantly, the results, obtained by the two completely different approaches, western blot and mass spectrometry, were concordant. Thus, western blot analysis confirmed the specific binding of fibronectin and C1qB to rAAV-8, the preferential binding of vitronectin and GPX3 to rAAV-9, and the equal interactions of PF4 and clusterin with both serotypes.

### Proteins Interacting with rAAV-8 and rAAV-9 in Human Serum

The list of proteins interacting with rAAV-8 and rAAV-9 in human serum is presented in Table 3. Similar to the results obtained for rAAV-8 and rAAV-9 in mouse serum, no majority protein interacting with these serotypes was observed in human serum. Overall, 34 proteins met the selection criteria for rAAV-8, and 26 proteins for rAAV-9, with 19 proteins being in common to both serotypes. These in-common proteins account for more than 80% of the bound proteins in both serotypes. The top four most abundant proteins bound by rAAV-8 (PF4, ITIH2, fibronectin, and ITH1) and the top four for rAAV-9 (vitronectin, PF4, prothrombin, and complement C4-B) accounted for 50% of their bound proteins (Table 3).

**Table 3:** Proteins in Human Serum Interacting with rAAV-8 and rAAV-9

AAV-8			AAV-9		
Description	% of Bound Proteins^a^	n^b^	Description	% of Bound Proteins^a^	n^b^
Platelet factor 4^c^	13.8	5	Vitronectin^c^	17.7	6
Inter-alpha-trypsin inhibitor heavy chain H2^c^	13.5	6	Platelet factor 4^c^	15.1	8
Fibronectin^c^	11.7	5	Prothrombin^c^	9.6	6
Inter-alpha-trypsin inhibitor heavy chain H1^c^	9.6	5	Complement C4-B	7.1	5
Apolipoprotein E^c^	9.2	4	Plasminogen^c^	7.0	4
Gelsolin	4.5	4	Inter-alpha-trypsin inhibitor heavy chain H2^c^	5.9	5
Complement C1q subcomponent subunit C^c^	4.0	7	Complement C4-A	5.6	5
Complement factor B	3.9	5	Apolipoprotein E^c^	5.2	4
Inter-alpha-trypsin inhibitor heavy chain H4^c^	3.7	7	Hyaluronan-binding protein 2	3.9	8
Vitronectin^c^	3.1	6	Clusterin^c^	3.5	5
Complement C1q subcomponent subunit B^c^	3.0	7	Inter-alpha-trypsin inhibitor heavy chain H1^c^	3.5	5
Plasminogen^c^	2.8	5	Inter-alpha-trypsin inhibitor heavy chain H4^c^	2.8	6
Alpha-1B-glycoprotein	1.8	5	Fibronectin^c^	2.6	5
Protein AMBP^c^	1.6	4	Histidine-rich glycoprotein^c^	2.5	6
Inter-alpha-trypsin inhibitor heavy chain H3	1.6	4	Complement C1q subcomponent subunit C^c^	2.5	6
Complement C1q subcomponent subunit A	1.6	5	Complement C1q subcomponent subunit B^c^	1.7	5
Clusterin^c^	1.6	4	Protein AMBP^c^	0.9	5
Complement C5	1.3	5	Ceruloplasmin^c^	0.6	4
Myeloperoxidase^c^	1.2	7	Antithrombin-III^c^	0.5	4
Complement component C9	1.1	5	Plasma serine protease inhibitor	0.5	7
Plasma protease C1 inhibitor	1.0	4	Selenoprotein P^c^	0.3	6
Ceruloplasmin^c^	1.0	7	Alpha-2-antiplasmin^c^	0.3	7
Prothrombin^c^	0.8	4	Nidogen-1	0.3	6
Histidine-rich glycoprotein^c^	0.7	4	Myeloperoxidase^c^	0.3	6
Complement C1r subcomponent	0.6	4	Angiotensinogen	0.1	4
Complement component C8 beta chain	0.3	4	–	–	–
Pigment epithelium-derived factor	0.2	5	–	–	–
Thrombospondin-1	0.2	6	–	–	–
Galectin-3-binding protein	0.1	4	–	–	–
Fibulin-1	0.1	4	–	–	–
Alpha-2-antiplasmin^c^	0.1	4	–	–	–
Antithrombin-III^c^	0.1	4	–	–	–
Carbonic anhydrase 1	0.1	4	–	–	–
Selenoprotein P^c^	0.1	6	–	–	–
In common with AAV9	81.3		In common with AAV8	82.5	–

a The sum of protein quantities meeting the selection criteria for the vectors was considered as 100%; the relative quantity of each protein is expressed as a % of the sum. Proteins are arranged in the order of decreasing quantities.

b n is the number of experiments where a protein was detected. Seven independent experiments were performed with rAAV-8 and eight experiments with rAAV-9.

c Proteins are common between rAAV-8 and rAAV-9.

**Figure 3:**
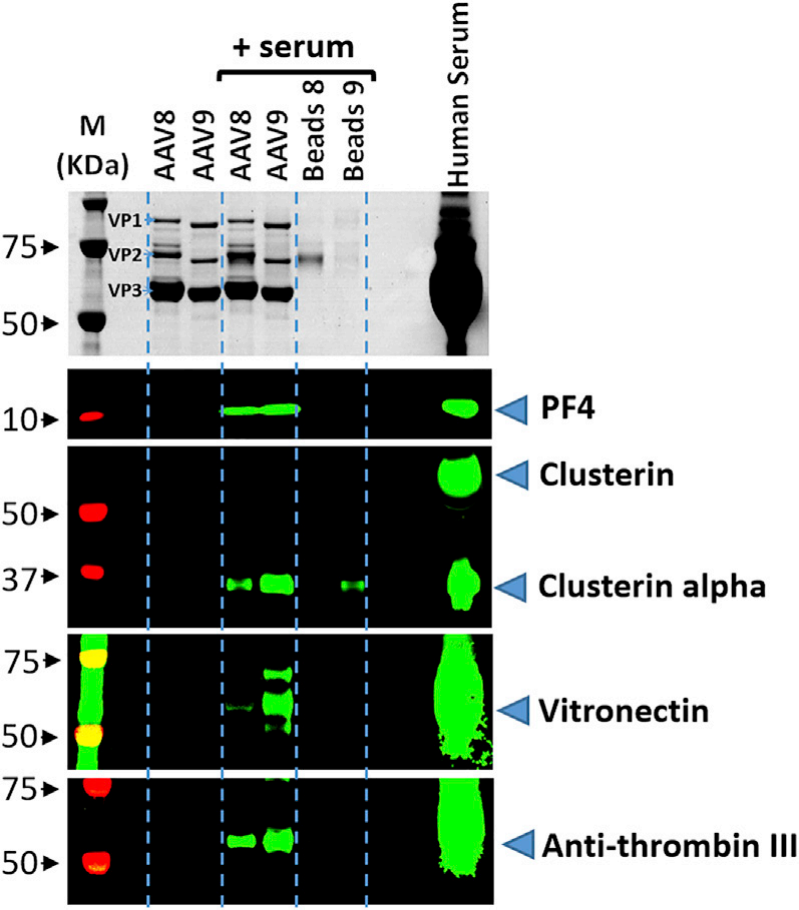
Western Blot Analysis of Proteins Binding to rAAV-8 and rAAV-9 in Human Serum Upper panel: Coomassie staining (VP1, VP2, and VP3 indicate position of the respective vector proteins); lower panel: western blot analysis of selected proteins binding to rAAV-8 and rAAV-9. From left to right: M, molecular weight (MW) markers; AAV8, AAV9, respective immobilized vectors without incubation with serum; AAV8, AAV9 (serum), respective immobilized vectors after incubation with serum; beads 8, beads 9 (serum), serum proteins retained by the empty beads used for the respective serotype immobilization; serum, deposition of 1 μL of serum.

Interactions of PF4, clusterin, antithrombin III, and vitronectin with rAAV-8 and -9 were confirmed by western blot analysis (Figure 3). Importantly, the relative quantities of proteins interacting with rAAV-8 or -9 visualized by western blot analysis were in agreement with those estimated by mass spectrometry. Thus, both methods detected nearly equal quantities of PF4 bound to rAAV-8 and rAAV-9, and stronger binding of clusterin, anti-thrombin III, and vitronectin to rAAV-9.

### Interaction of Human Serum Albumin with rAAV-8

**Figure 4:**
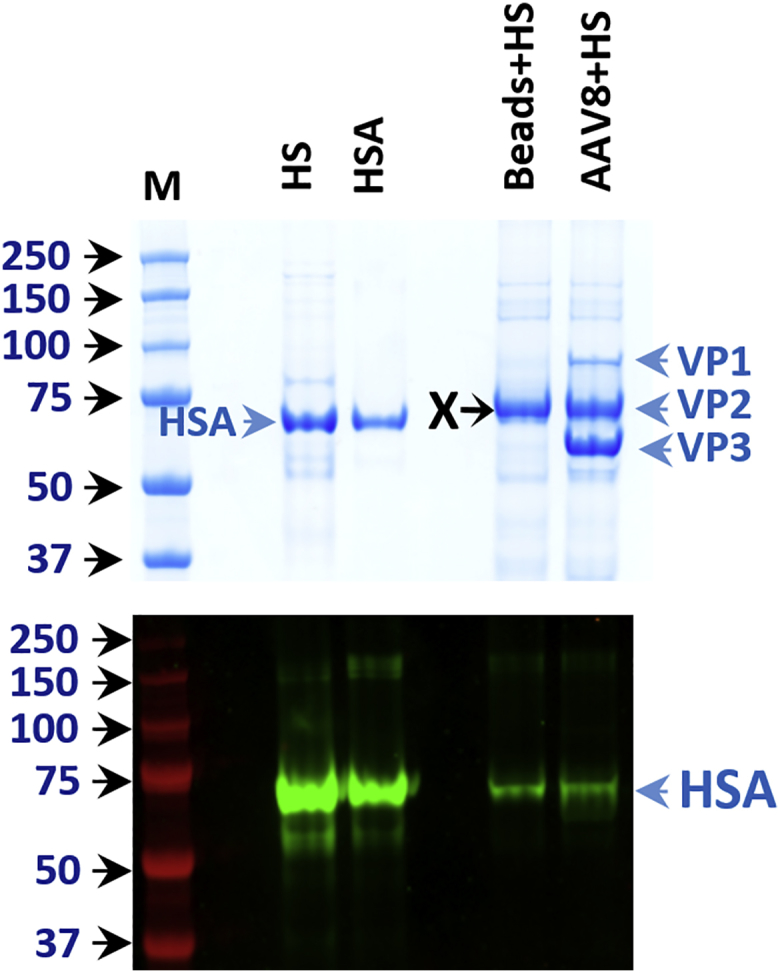
Western Blot Analysis of Human Albumin Binding to rAAV-8 in Serum Upper panel: Coomassie staining (VP1, VP2, and VP3 indicate position of the respective vector proteins; VP2 migrates at the same position as non-specifically bound protein X [this protein was identified by MALDI-TOF spectrometry as the 68-kDa C4b-binding protein alpha chain]; HSA indicates the position of human serum albumin). Lower panel: western blot analysis of HSA binding to rAAV-8. From left to right: M, molecular weight (MW) markers; HS, 0.1 μL of human serum; HSA, 1 μg of purified human albumin; Beads+HS, empty beads after incubation with human serum; AAV8+HS, immobilized rAAV-8 after incubation with human serum. HSA binds at the same low level to empty beads and rAAV-8-beads, demonstrating absence of specific binding of HSA to rAAV-8.

Recently, interactions between human serum albumin (HSA) and rAAV-8 have been described.^12^ Under the binding conditions of our assays, we did not detect any interactions between human or mouse albumins with any of the studied serotypes. In order to confirm our data, we performed additional experiments, where specific binding of HSA to rAAV-8 in serum was tested by western blot analysis after co-precipitation. Because there was no detailed description of the washing conditions used by Wang et al.,^12^ we used mild washing conditions (five 3-min washings in 1× PBS). Confirming our initial data, no specific binding of HSA to rAAV-8 was observed (Figure 4). A supplementary band of ∼70 kDa, which non-specifically bound to empty beads, migrated at the same position as VP2 of rAAV-8; it was identified by MALDI-TOF as the 68-kDa C4b-binding protein alpha chain. Importantly, in Wang et al.’s^12^ work, HSA enhanced AAV transduction in a mouse model of hemophilia. We suggest that the *in vivo* effects seen in Wang et al.’s^12^ experiments could have been provoked by other mechanisms, as, for example, an early response to HSA that changed vascular permeability.

### Bioinformatics Analysis of Bound Proteins

**Figure 5:**
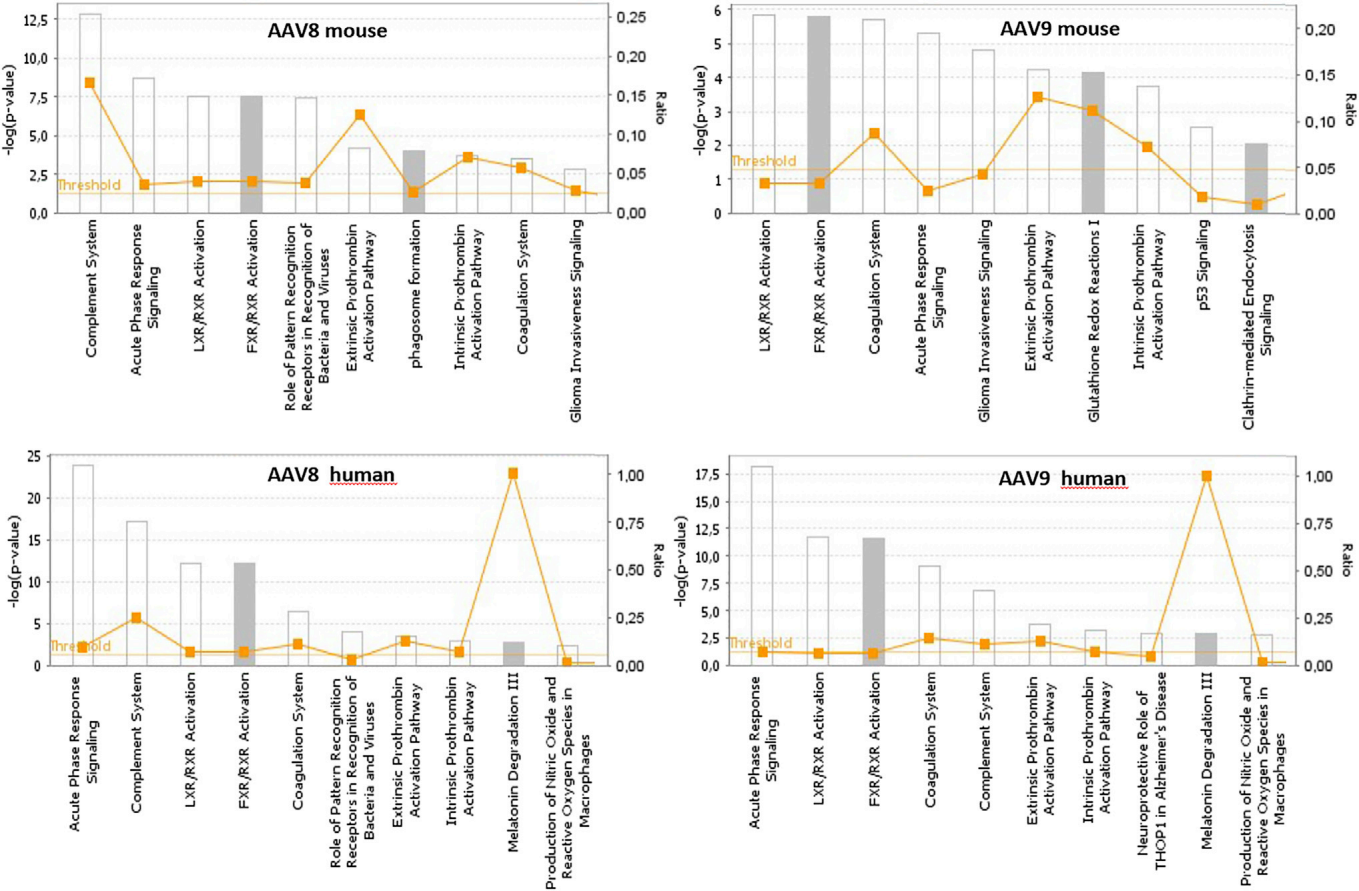
Ingenuity Pathway Analysis of the Proteins Interacting with rAAV-8 and rAAV-9 Serotypes in Mouse and Human Sera Significantly overrepresented canonical pathways are presented according to their p value (-Log), as calculated by ingenuity pathway analysis (IPA). Ratio, orange squares: the ratio of listed proteins found in each pathway over the total number of genes in that pathway. The threshold line (yellow) corresponds to a p value of 0.05. The p value was calculated using the right-tailed Fisher’s exact test.

To map our findings to biological processes, i.e., to determine disease states that would enhance or diminish the levels of these interacting proteins, we analyzed the identified proteins using the Ingenuity Pathway Analysis Tool (https://www.ingenuity.com). The results of this analysis demonstrated that the mouse and human sera proteins interacting with rAAV-8 and rAAV-9 are implicated in acute-phase response signaling, the complement system, coagulation, and the canonical liver X receptor (LXR)/retinoid X receptor (RXR) and farnesoid X receptor (FXR)/RXR pathways (Figure 5). LXR/RXR is involved in the regulation of lipid metabolism, inflammation, and cholesterol-to-bile acid catabolism. Along with RXR, FXR plays a crucial role in linking bile acid regulation with lipoprotein, lipid, and glucose metabolism.

Even though the pathways recognized by the Ingenuity analysis indicate protective roles for the identified interactive proteins, viruses have adopted numerous mechanisms to evade the destructive effects of the host’s defense systems, sometimes even using binding to the elements of these systems to increase their invasive efficacy (for a review, see Mullick et al.^13^ and Blue et al.^14^). To evaluate the *in vivo* effects of individual serum proteins binding to the vectors, one needs the respective KO mouse models.

### Effect of mPF4 on rAAV-8 and rAAV-9 Efficacy *In Vivo*

**Figure 6:**
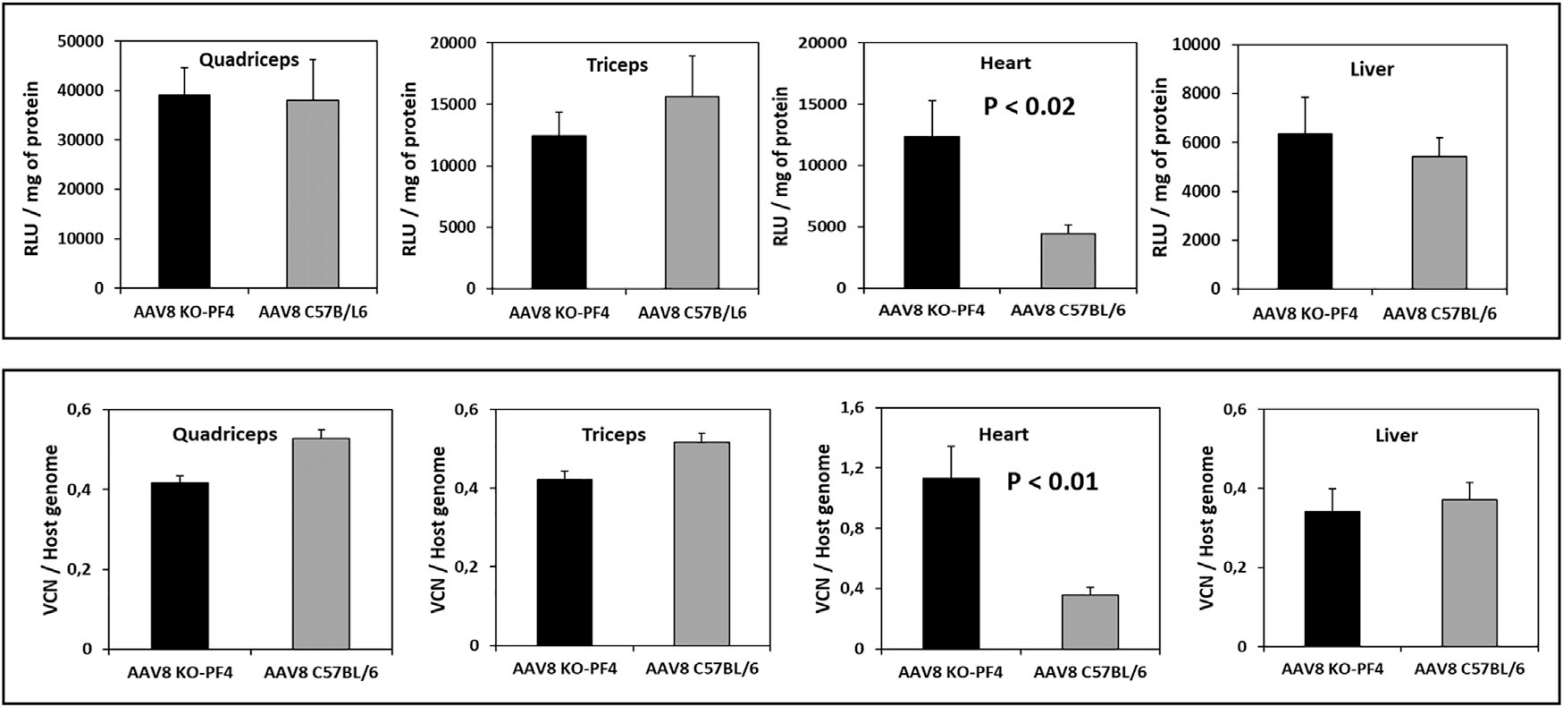
Effects of PF4 on rAAV-8 Transduction Efficacy C57BL/6 and C57BL/6KO-PF4 mice received intravenous injections of rAAV-8 (1.5 × 10e11 viral genomes) encoding for luciferase; 2 weeks after administration, luciferase activity (upper panel) and vector copy numbers (lower panel) were estimated in different organs. The measurements by both methods demonstrated a 2.8-fold decrease in rAAV-8 transduction efficacy to the heart. Levels of significance were determined using the Student’s t test. The data are presented as mean values ± SEM. Eight mice per group were used in this experiment; results are representative of two independent experiments.

**Figure 7:**
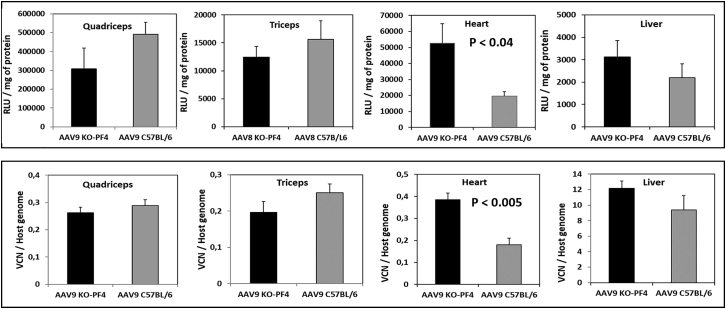
Effects of PF4 on rAAV-9 Transduction Efficacy C57BL/6 and C57BL/6-KO-PF4 mice received intravenous injections of rAAV-9 (1.5 × 10e11 viral genomes) encoding for luciferase; 2 weeks after administration, luciferase activity (upper panels) and vector copy numbers (lower panels) were estimated in different organs. The presence of PF4 decreased rAAV-9 efficacy in the heart by 2-fold. Levels of significance were determined using the Student’s t test. The data are presented as mean values ± SEM; four mice were used for each condition. Results are representative of two independent experiments.

Given that PF4 from mouse or human sera binds rAAV-8 and rAAV-9 equally well, we chose this protein for our *in vivo* studies. To establish the role of mPF4 on the vectors’ transduction efficacy, we used the mPF4-KO model on a C57BL/6 genetic background.^15^, ^16^, ^17^ Wild-type and PF4-deficient C57BL/6 mice received intravenous injections of either of the two rAAV vectors containing a gene encoding luciferase. Two weeks after vector administration, luciferase activity and viral genome/vector copy numbers were evaluated in the quadriceps, triceps, heart, and liver (Figures 6 and 7). In control experiments, mice were injected with an rAAV-6 vector that does not interact with PF4.

Corroborating previously published results,^18^ both vectors demonstrated high efficacy for muscle and heart transduction. Importantly, the presence of mPF4 in the blood of wild-type mice diminished the level of heart transduction by 2- to 3-fold for both vectors. No statistically significant differences in vector copy numbers or luciferase activity were found for the quadriceps, triceps, or liver between the wild-type and PF4-deficient mice (Figures 6 and 7). No effect of PF4 on rAAV-6 efficacy was observed in the control experiments (data not shown), thus confirming that the decrease of heart transduction in wild-type mice was really due to interactions of rAAV-8 and rAAV-9 vectors with mPF4.

### Effect of huPF4 on rAAV-8 and rAAV-9 Efficacy *In Vivo*

**Figure 8:**
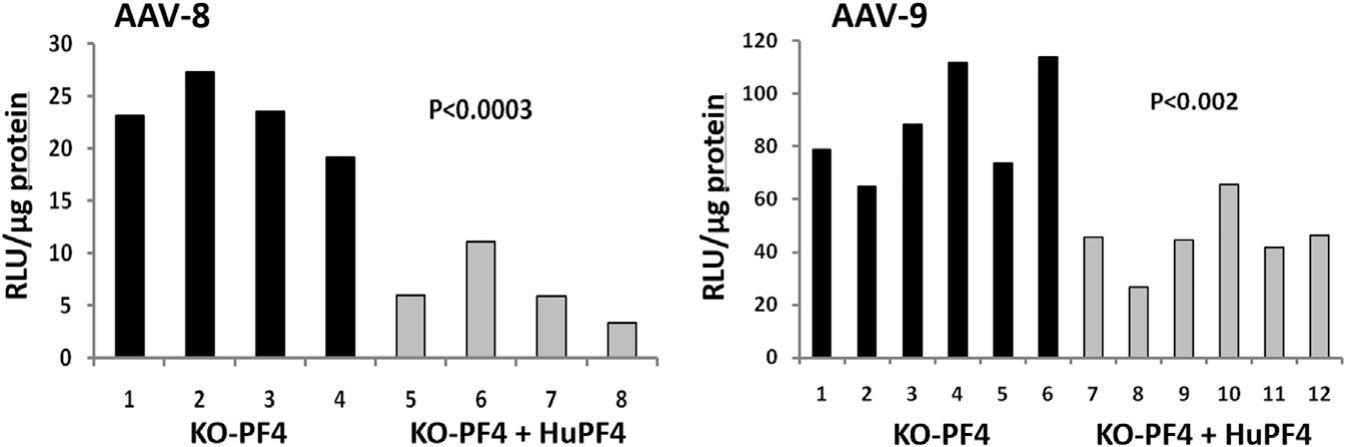
Decrease in Heart Transduction with rAAV-8 and rAAV-9 When huPF4 Is Present 1.5 × 10e11 viral genomes of the vectors were pre-incubated with mouse serum from C57BL/6 PF4 knockout mice expressing human PF4 (KO-PF4 + huPF4, gray bars), or from C57BL/6 PF4 knockout mice (KO-PF4, black bars), and then injected into the tail vein of C57BL/6 PF4 knockout mice. The data are presented as individual measurements for each animal. Levels of significance were determined using Student’s t test.

To determine the role of huPF4 protein on rAAV-8 and rAAV-9 transduction efficacy *in vivo*, we used the mouse C57BL/6 PF4 KO model engineered to express huPF4 protein.^15^, ^16^, ^17^ Because the level of PF4 *in vivo* depends on platelet activation,^19^ and that varies greatly with inflammatory state,^20^ we pre-incubated the rAAV-8 and rAAV-9 vectors coding for luciferase before intravenous injection with either serum from mice expressing huPF4 or serum from C57BL/6 PF4 KO mice. The concentration of huPF4 in the serum of the C57BL/6 mouse-PF4 KO model expressing huPF4 was equal to 10 μg/mL, which corresponds to the normal PF4 concentration in human serum.^21^ The impact of huPF4 on vector efficacy was estimated by comparing luciferase expression, 2 weeks post-injection, in the liver, quadriceps, and heart of the mice. In these experiments, we reproducibly obtained a 2-fold decrease in heart transduction when huPF4 was present (Figure 8), whereas no statistically significant difference in other studied organs was observed.

## Discussion

Achieving a level of clinical relevance for gene therapy using recombinant adeno-associated vectors (rAAV) usually requires widespread distribution of the vector, for which systemic delivery by intravenous injection is the optimal route of administration. Several naturally occurring AAV serotypes and variants have been used as gene therapy vectors. The difference in tissue tropism of these vectors, and the capacity to evade preexisting neutralizing antibodies, depends on the makeup of the vector capsid.^22^ Importantly, although studies have identified the primary receptors and co-receptors for rAAVs 1–6, 8, and 9 for cells in culture,^23^, ^24^, ^25^, ^26^, ^27^, ^28^ their relative contributions for *in vivo* vector efficacy are less clear. We have recently shown that interactions with species-specific blood proteins drastically change rAAV-1 and rAAV-6 biodistribution and efficacy.^9^, ^10^ In the present study, we have identified serum proteins interacting with AAV-8 and -9, two serotypes often used in preclinical and clinical transgenic studies, especially in the field of neuromuscular diseases.

### Validation of an Assay for Identification of Proteins Interacting with Vectors

Progress in protein identification by mass spectrometry approaches permits us to identify thousands of proteins per experiment, thus giving us the ability to analyze co-precipitated proteins without preliminary separation or even elution from the carrier. In the present work, we took advantage of the high-resolution power of proteomics to introduce a new assay for characterization of serum proteins bound to rAAV vectors. In this assay, the serum proteins bound to vector capsids immobilized on beads were directly digested by trypsin and then identified from their digest peptides by mass spectrometry. Two parameters, specificity, which compares the quantity of a protein bound to rAAV-beads versus empty beads, and reproducibility, which is an account of the number of experiments where a protein was detected, permitted us to distinguish proteins specifically bound to the vector from non-specifically bound (bound to the beads used to immobilize the vectors). Importantly, the pertinence of this approach was validated by identification of mCRP and huG3BP as major factors interacting with rAAV-6 in their respective sera. This approach, being more sensitive than the one previously applied,^9^, ^10^ permitted us to identify several additional proteins interacting with rAAV-6 in human and mouse sera. These new proteins bind rAAV at much lower levels compared with mCRP or huG3PB, and their roles in altering biodistribution and efficacy *in vivo* remain elusive. Absence of competition for binding with rAAV-6 between mCRP and vitronectin reasonably suggests that there are different sites on the rAAV surface that are responsible for interactions with at least these two different serum proteins.

### Proteins Interacting with rAAV-8 and rAAV-9

Maximum-likelihood phylogeny, relating to the 75 isolates of AAV, indicates that rAAV-8 and rAAV-9 are relatively close serotypes, which are quite different from rAAV-1 and rAAV-6.^29^ These serotypes utilize different cellular receptors for cellular attachment: although both rAAV-1 and rAAV-6 bind N-linked sialic acid (and rAAV-6 also binds heparin sulfate proteoglycan),^3^, ^30^ rAAV-8 and rAAV-9 bind to the laminin receptor (rAAV-9 also binds N-linked galactose),^23^, ^27^, ^31^ and they demonstrate different efficacy and biodistribution *in vivo*.^31^ Importantly, during the next step of transduction, cellular entry, different AAV serotypes use different domains of the universal AAV cellular entry receptor (adeno-associated virus receptor [AAVR]).^32^, ^33^ It would not be surprising if serum components interacting with rAAV-1 and rAAV-6 from one side, and rAAV-8 and rAAV-9 from another, were different. Indeed, in the present study, we show that these serotypes interact in an entirely different manner with serum proteins; while there is a one major serum protein that binds rAAV-1 or rAAV-6, rAAV-8 and rAAV-9 bind more than 10 or 20 proteins in mouse and human sera, respectively (Tables 2 and 3).

Interestingly, there are nine and six proteins that are in common between human and mouse proteins bound to rAAV-8 and rAAV-9, respectively (Table 4). These nine in-common proteins represent 51% of the bound proteins in mouse serum and 40% in human serum, in the case of rAAV-8. The six in-common proteins represent 86% of the bound proteins in mouse serum and 51% in human serum for rAAV-9. If the *in vivo* effects of proteins identified in the present study were proportional to their percentage in the total protein content, then the substantial similarity in quantitative protein composition bound to rAAV-8 and rAAV-9 in mouse and human sera predicts that there will be similar behavior of these two vector serotypes (-8 and -9) in mouse and human species.

**Table 4:** In-Common Proteins Interacting with rAAV-8 and rAAV-9 in Mouse and Human Sera

	% of Bound Proteins	
Description	Mouse Serum	Human Serum
AAV-8		

Platelet factor 4	15.70	13.80
Vitronectin	10.30	3.10
Complement C1q subcomponent subunit A	5.90	1.60
Complement C1q subcomponent subunit B	5.50	3.00
Thrombospondin-1	5.30	0.20
Complement C1q subcomponent subunit C	5.20	4.00
Fibronectin	4.90	11.70
Clusterin	4.60	1.60
Prothrombin	4.50	0.80
% of total	51.60	39.80

AAV-9		

Histidine-rich glycoprotein	25.20	2.50
Platelet factor 4	19.20	15.10
Vitronectin	18.50	17.70
Prothrombin	6.90	9.60
Inter-alpha-trypsin inhibitor, heavy chain 4	6.50	2.80
Clusterin	5.00	3.50
% of total	86.30	51.20

In-common proteins in mouse and human sera bound to both rAAV-8 and rAAV-9. Data are compiled from Tables 3 and 4.

Analysis of these proteins by the Ingenuity Pathway Analysis Tool demonstrated that in mouse and human sera, proteins interacting with rAAV-8 and rAAV-9 are involved in acute-phase response signaling and complement system canonical pathways (Figure 5). Even though pathways recognized by the Ingenuity analysis indicate protective roles for the identified proteins, viruses have evolved numerous mechanisms to evade the host’s defenses.^34^ Moreover, some viruses can exploit the complement system to promote infection, either by binding directly to complement receptors (CRs) to gain entry to host cells or indirectly through complement-opsonized virus interactions.^35^, ^36^, ^37^, ^38^ Interaction of rAAV-1 and rAAV-6 with CRP in mouse serum is another example of enhancement of viral vector efficacy by the host defense system.^9^, ^10^

### Interactions with PF4 Affect rAAV-8 and rAAV-9 Efficacy *In Vivo*

Platelet factor-4, also known as CXCL4, is one of the most abundant platelet chemokines; it is released in micromolar concentrations from the platelets’ alpha granules upon platelet activation and is known for its pleiotropic biological functions.^39^ It belongs to the PF-4 family of kinocidins, with a relatively large spectrum of antimicrobial activities.^40^ Accumulating evidence suggests that, unlike other chemokines that bind to specific receptors, PF4’s biology depends on its unusually high affinity for heparin sulfates and other negatively charged molecules. It was proposed that the main biological role of PF4 is to neutralize surface heparin sulfate side chains of glycosaminoglycans and to optimize thrombus development at sites of vascular injury. Additionally, PF4 binds to several other proteins that are central to thrombosis, angiogenesis, and atherogenesis. These interactions may also contribute to its biological and pathological effects.^41^ Surprisingly, the presence of PF4 in mouse serum leads to a specific decrease of rAAV-8 and rAAV-9 efficacy in the heart, whereas transduction of other tested organs was independent of PF4’s presence. We suggest that the binding of PF4 could mask some determinants on the vector’s surface that are more important for heart transduction than for transduction of other organs, or more likely, that the targeted heart cells shuttle PF4-bound vectors through intracellular compartments, such as lysosomes, that do not lead to transduction and/or expression. Importantly, the impact of PF4 on rAAV-8 and rAAV-9 efficacy was substantially lower compared with the effects of “dominant” proteins CRP and G3BP on rAAV-6 and rAAV-1 efficacy in their respective models.^9^, ^10^ The relatively low impact of huPF4 on rAAV-8 and rAAV-9 efficacy could be linked to the fact that PF4 is only one of many proteins interacting with these vectors in mouse serum, and its effects could be proportional to its content in the bulk of proteins interacting with the vector.

Taken together, the present findings indicate that vector capsid interactions with serum proteins can be crucial parameters when translating results obtained with animal models to human reactions. Fortunately, our results favor the hypothesis that the impact of serum proteins on the efficacy obtained with mouse models for rAAV-8 and rAAV-9 using systemic delivery will be equivalent to that for humans.

## Materials and Methods

### rAAV Production

Adenovirus-free vectors were generated either by using a three-plasmid transfection of HEK293 cells^42^, ^43^ (two batches of rAAV-8 and three batches of rAAV-9) or by double infection of Sf9 cells^44^ (two batches of AAV-8 and two batches of AAV-9). Vectors were purified by affinity chromatography using AVB Sepharose HP (AAV-6, AAV-8) (GE Healthcare Life Sciences, Piscataway, NJ, USA) or POROS CaptureSelect AAV9 Affinity Resin (AAV-9) (Thermo Fisher).

The number of viral genomes was estimated by qPCR of extracted vector DNA. The number of vector physical particles was estimated either by an ELISA-based method or by quantification of VP3 protein after SDS-PAGE analysis stained with Coomassie G250, with BSA as a standard. The content of full capsids was very similar for the different serotypes used in this study: the ratio of viral genomes to physical particles varied from 1/3 to 1/10.

### Vector-Protein Binding Assay

Co-precipitation of blood proteins was performed with rAAV-6 and -8 vectors immobilized on AVB Sepharose HP beads, as described previously,^9^ and with rAAV-9 immobilized on POROS CaptureSelect AAV9 Affinity Resin. Immobilized rAAV vectors (10 μL of beads with 1 × 10e11 vector particles) were incubated with 100 μL of serum from either species for 1 hr. Beads were collected by centrifugation and washed four times with 1× PBS, 0.5% Triton X-100. The precipitate was either digested with trypsin for mass spectrometry analysis or dissolved in the Laemmli sample buffer for further analysis by western blot.

To avoid an impact of a particular serum batch on the results, different sources of serum were used in the study. Human serum was either from a commercial source (Sigma, St. Louis, MO, USA) or was from serum samples from healthy human adults obtained in accordance with regulatory guidelines. Mouse serum came from four independent sources: mice C57BL/6 (Charles River, France), commercial serum from SIGMA (S3509), and non-Swiss Albino mouse serum and BALBC mouse serum (IMS-SER and IGMS-BC-SER, respectively; Innovative Research). Sera used in this study were assessed for the presence of antibodies according to Boutin et al.^45^ and were seronegative for respective AAV serotypes. Before co-precipitation, endogenous antibodies were depleted from all the sera by incubation with Pierce Protein A/G Plus agarose (Thermo Scientific). All procedures involving animals were performed according to the guidelines of our Institute’s Animal Ethics Committee.

### Animals

Healthy, 4-week-old C57BL/6 male mice, C57BL/6 PF4-KO mice, and C57BL/6 PF4-KO mice expressing 10 μg/mL huPF4^15^, ^16^, ^17^ were used in this study. C57BL/6 mice were obtained from Charles River (France). C57BL/6 mPF4^−/−^ mice were maintained by homozygote breeding. To obtain mouse serum containing huPF4 (heterozygote mouse C57BL/6 huPF4^+/−^^17^), heterozygote C57BL/6 huPF4^+/−^ mice were bred with C57BL/6 PF4 KO mice, and heterozygote newborn mice were identified by western blot analysis using anti-huPF4 antibody. All experiments were performed independently at least three times, and each experiment comprised four to five mice for each condition. Results, if not otherwise stated, are given as mean ± SD. For systemic delivery, rAAV expression vectors coding for firefly luciferase under the cytomegalovirus (CMV) promoter were injected into the lateral tail vein. Two weeks after injection, luciferase expression was evaluated by measuring luciferase protein activity and vector copy numbers in tissue extracts.

To test the impact of loss of mPF4 or the presence of huPF4 on rAAV-9 efficiency and distribution in C57BL/6 PF4 KO mice, before the injections we incubated 1.5 × 10e11 viral genomes of rAAV with either 200 μL of serum from C57BL/6 PF4 KO mice or 200 μL of serum from C57BL/6 PF4 KO mice expressing huPF4,^15^ for 1 hr at ambient temperature.

### *Ex Vivo* Biodistribution

#### Luciferase Activity

Mice were sacrificed 14 days after rAAV injection, and the liver, heart, kidney, quadriceps, and triceps muscles were recovered. Luciferase activity was analyzed from 50 mg of tissue samples homogenized in 250 μL of lysis buffer (12.5 mM Tris-phosphate [pH 7.8], 7.5% glycerol, 0.05 M EDTA, 10 mM DTT, 4 mM MgCl_2_, and 1% Triton X-100). Homogenates were centrifuged at 18,000 × *g* for 10 min at 4°C; 20 μL of each supernatant was used in duplicate reactions to measure luminescence with a Wallac VICTOR 2 luminometer (Perkin Elmer/Life Sciences, Waltham, MA, USA). Luminescence was acquired over a 10-s interval after simultaneous addition of 2 μM ATP in 100 μL of the lysis buffer without Triton X-100 and 100 μL of 170 mM D-Luciferin. The Bradford assay was used to determine protein concentration. Luciferase levels are shown as relative light units normalized to protein content.

### DNA Isolation and Real-Time PCR

Homogenized samples were mixed with 1% SDS and 400 μg/mL proteinase K and digested overnight to determine the numbers of AAV genome copies in injected mice. DNA was isolated by the phenol/chloroform/isoamyl alcohol (IAA) method, and DNA concentration was estimated with a NanoDrop Spectrophotometer (Thermo Scientific). One hundred nanograms of DNA was analyzed by qPCR on an ABI 7900HT (Applied Biosystems) according to the manufacturer’s instructions. Primer pairs and 6-FAM 5′ end and TAMRA 3′ end-labeled probes were designed for the CMV promoter region of the vector and for the endogenous *titin* gene, as follows: CMV F: 5′-CATCAATGGGCGTGGATAGC-3′, R: 5′-GGAGTTGTTACGACATTTTGGAAA-3′; CMV Probe: 5′-6-FAM-ATTTCCAAGTCTCCC-TAMRA-3′; *Titin* F: 5′-AAAACGAGCAGTGTGAGC-3′, R: 5′-TTCAGTCATGCTGCTAGCGC-3′; and *Titin* Probe: 5′-6-FAM-ACGGAAGCGTCTCGTCTCAGTC-TAMRA-3′.

### Identification and Quantification of Proteins by Mass Spectrometry

For mass spectrometry analysis, co-precipitated proteins were directly digested by trypsin in 50 μL of buffer containing 100 mM ammonium carbonate (pH 8.0) and 500 ng of trypsin (Sequence Grade Trypsin; Promega) for 16 hr at 37°C and stored at −20°C until use. The peptide mixture was desalted using a ZipTip μ-C18 Pipette Tip (Millipore) and separated with an Easy nano-LC Proxeon system (Thermo Fisher Scientific) equipped with a reverse-phase C18 column (Easy-Column Proxeon C18, L 15 cm, ID 75 μm). Eluates were monitored by a LTQ Velos Orbitrap mass spectrometer (Thermo Fisher Scientific) and tandem MS (MS/MS). Data were processed with Proteome Discoverer 1.4 software (Thermo Fisher Scientific) coupled to an in-house Mascot Search Server (Matrix Science, 2.3.2, version 213) using the SwissProt database, as described previously.^46^ The relative abundance of each protein identified in serum from Duchenne muscular dystrophy (DMD) cases or healthy patients was estimated by label-free quantification using Progenesis LC MS software (Nonlinear Dynamics, 4.0 version). Average normalized abundances (ANA), reflecting the relative quantities of proteins by Progenesis analysis, were used to compare quantities of protein bound to a vector.

#### Western Blot

Protein samples were separated by SDS-PAGE electrophoresis (1.0 mm, 4%–12% gradient, Novex NuPAGE Bis-Tris Gel; Life Technologies) and transferred onto a Protran Premium nitrocellulose membrane (GE Healthcare). The primary antibodies used in this study are listed in Table S1. After incubation with corresponding secondary IRDye-800CW-conjugated antibodies (1:10,000; LI-COR Biosciences), infrared fluorescence was read on an Odyssey Imaging System (LI-COR Biosciences). Band intensities were measured using Odyssey application software (Image Studio Lite, version 4.0; LI-COR Biosciences).

## Author Contributions

F.S., J.D., J.-M.C., M.P.L., and L.G. designed the research; J.D., T.L., C.G., G.G., and C.J. performed the research; J.R. analyzed the data; F.S. and L.G. wrote the paper.

## Supplemental Information

Document S1. Table S1

Document S2. Article plus Supplemental Information
