# Supplementary material for: AAV-8 and AAV-9 Vectors Cooperate with Serum Proteins Differently Than AAV-1 and AAV-6
Source: Mol Ther Methods Clin Dev. 2018 Aug 8;10:291–302. doi: 10.1016/j.omtm.2018.08.001 (PMC6111067; doi:10.1016/j.omtm.2018.08.001)
Supplement: Document S1. Table S1 [file mmc1.pdf]

OMTM, Volume *10*

Supplemental Information

AAV-8 and AAV-9 Vectors Cooperate with Serum Proteins Differently Than AAV-1 and AAV-6

Jérôme Denard, Jérémy Rouillon, Thibaut Leger, Camille Garcia, Michele P. Lambert, Graziella Griffith, Christine Jenny, Jean-Michel Camadro, Luis Garcia, and Fedor Svinartchouk

Supplemental Information

Supplemental Table 1. Primary antibodies used in the study

Protein	Catalog number	Company name	Dilution for WB
**Human**			
Histidine-rich glycoprotein (HRG)	AF1905	R&D	1:1000
Clusterin	Sc-6419	Santa Cruz	1:500
Vitronectin	Sc-28929	Santa Cruz	1:1000
Platelet factor 4 (PF4)	Sc-374195	Santa Cruz	1/1000
Glutathione peroxidase 3 (GPX-3)	AF4199	R&D	1:1000
Complement component 1q subcomponent B (C1qB)	PA5-35369	Pierce	1 :1000
Fibronectin	Sc-6952	Santa Cruz	1:1000
**Mouse**			
Platelet factor 4 (PF4)	AF595	R&D	1:1000
Clusterin	Sc-6419	Santa Cruz	1:500
Vitronectin	Sc-28929	Santa Cruz	1:1000
Anti-thrombin III	Ab126598	Abcam	1:1000
